# Toward tailored care for families with multiple problems: A quasi‐experimental study on effective elements of care

**DOI:** 10.1111/famp.12745

**Published:** 2021-12-21

**Authors:** Loraine Visscher, Sijmen A. Reijneveld, Jana Knot‐Dickscheit, Tom A. van Yperen, Ron H.J. Scholte, Marc J.M.H. Delsing, K. Els Evenboer, Danielle E.M.C. Jansen

**Affiliations:** ^1^ Department of Health Sciences University Medical Center Groningen University of Groningen Groningen The Netherlands; ^2^ Department of Child and Family Welfare University of Groningen Groningen The Netherlands; ^3^ Behavioural Science Institute Radboud University Nijmegen The Netherlands; ^4^ Praktikon Nijmegen The Netherlands; ^5^ Department of Healthy Society Windesheim University of Applied Sciences Zwolle The Netherlands; ^6^ Department of Sociology and Interuniversity Center for Social Science Theory and Methodology (ICS) University of Groningen Groningen The Netherlands

**Keywords:** child and adolescent social care, families with multiple problems, interventions, practice elements, program elements

## Abstract

Several effective interventions have been developed for families with multiple problems (FMP), but knowledge is lacking as to which specific practice and program elements of these interventions deliver positive outcomes. The aim of this study is to assess the degree to which practice and program elements (contents of and structure in which care is provided) contribute to the effectiveness of interventions for FMP in general and for subgroups with child and/or parental psychiatric problems, intellectual disabilities, or substance use. We performed a quasi‐experimental study on the effectiveness of practice and program elements provided in attested FMP interventions. Using self‐report questionnaires, we measured primary (child's internalizing and externalizing problems) and secondary (parental stress and social contacts) outcomes at the beginning, end, and three months thereafter. By means of Latent Profile Analysis, we identified groups of families receiving similar combinations of practice elements (“profiles”), and we calculated propensity scores. Next, we assessed how practice element profiles and program elements affected improvement in outcomes, and whether these effects were moderated by subgroup characteristics. We found three practice element profiles (explorative/supportive, action‐oriented, and their combination), which were equally effective. Regarding program elements, effects were enhanced by more frequent telephone contact between visits and more frequent intervision. Effectiveness of practice and program elements varied for specific FMP subgroups. Variations in the content of care for FMP do not affect its effectiveness, but variations in the structure of the care do. This finding can help to further improve effective interventions.

## Resumen

Se han desarrollado varias intervenciones eficaces para familias multiproblemáticas, pero se sabe poco acerca de qué elementos específicos de la práctica y de los programas de estas intervenciones producen resultados positivos. El objetivo de este estudio es evaluar el grado en el cual los elementos de la práctica y de los programas (los contenidos de la asistencia y la estructura en la cual se presta) contribuyen a la eficacia de las intervenciones para familias multiproblemáticas en general, y para subgrupos de padres o hijos con problemas psiquiátricos, discapacidades intelectuales o consumo de sustancias. Realizamos un estudio cuasiexperimental sobre la eficacia de los elementos de la práctica y de los programas proporcionados en intervenciones certificadas para familias multiproblemáticas. Utilizando cuestionarios de autoinforme, medimos los resultados primarios (los problemas de interiorización y de exteriorización de los niños) y secundarios (estrés de los padres y contactos sociales) al comienzo, al final y tres meses a partir de entonces. Por medio del análisis de perfiles latentes, identificamos grupos de familias que recibían combinaciones similares de elementos de la práctica (“perfiles”) y calculamos los puntajes de propensión. Después, evaluamos cómo los perfiles de los elementos de la práctica y los elementos del programa afectaron las mejoras en los resultados, y si estos efectos estuvieron moderados por las características de los subgrupos. Encontramos tres perfiles de elementos de la práctica (exploratorio/comprensivo, orientado a la acción y su combinación) que fueron igualmente eficaces. Con respecto a los elementos de los programas, los efectos mejoraron con el contacto telefónico más frecuente entre visitas y una intervisión más frecuente. La eficacia de los elementos de la práctica y de los programas varió según los subgrupos específicos de familias multiproblemáticas. Las variaciones en el contenido de la asistencia para las familias multiproblemáticas no afectan su eficacia, pero las variaciones en la estructura de la asistencia, sí. Estos resultados pueden ayudar a mejorar aún más las intervenciones eficaces.

## 摘要

针对有多元问题家庭（FMP）目前已经开发了一些有效的干预措施，但对于这些干预措施中哪些具体的做法和方案要素能带来积极的结果，还知之甚少。本研究的目的是评估实践和项目要素（提供护理的内容和结构）在多大程度上有助于对多元问题家庭（FMP）进行干预，以及对有儿童和/或父母精神问题、智力障碍或药物使用的亚群进行干预的有效性。我们进行了一项准实验性研究，针对经研究证实的FMP干预措施中所提供的实践和项目要素的有效性。主要采用自我报告问卷，在开始、结束和三个月后测量了主要结果（儿童的内化和外化问题）和次要结果（父母的压力和社会接触）。通过潜质分析，确定了接受类似实践要素组合的家庭群体（"概况 " ），并计算出倾向性分数。接下来，研究评估了实践要素概况和项目要素如何影响结果的改善，以及这些影响是否被亚组特征所调节。研究者发现三种实践要素（探索性/支持性、行动导向以及它们的组合）同样有效。关于项目要素，访问之间的电话联系和访问的频率提高了效果。实践的有效性和项目元素因特定的 FMP 子群体而异。FMP治疗内容的不同不影响疗效，但治疗方式的不同影响疗效。这一发现有助于进一步改善有效的干预措施。

## INTRODUCTION

Families with multiple problems (FMP) face a wide range of complex and persistent problems during their lifetime (Morris, 2013; Spratt & Devaney, 2009; Tausendfreund et al., 2016). These problems can include behavioral problems of the child, parenting problems, family conflicts, health, and financial problems—often co‐occurring (Bodden & Deković, 2016). Due to the complexity, persistency, and intergenerational nature of their problems, FMP often have a long history of care, as well as their own unique combination of problems and needs. Working with these families thus requires a flexible approach, tailored to the needs and wishes of specific families.

Several studies have assessed the effectiveness of interventions targeting FMP, with effects varying largely between interventions, between countries, and even between different studies on the same intervention (Evenboer et al., 2018). In general, studies have shown that interventions for FMP have positive effects on, for example, family functioning (Al et al., 2012), prevention of out of home placement (Al et al., 2012; Bezeczky et al., 2020), and behavioral problems (Asscher et al., 2013; van der Pol et al., 2017; Veerman & De Meyer, 2015). However, studies show effect sizes to differ depending on the study design (e.g., specific outcomes measure that was considered), the specific intervention provided and the target group (van Assen et al., 2020; Evenboer et al., 2018). Therefore, final conclusions on the effectiveness of these interventions are hard to make. An explanation for these divergent outcomes may lie in differences between the potentially effective elements provided within the interventions.

Generally, the effectiveness of care for FMP is determined by common and specific factors of care. Common factors are factors that contribute to the effectiveness of care regardless of the type of treatment or the target group (Lambert et al., 1994). These can be related to the client (e.g., level of motivation, commitment to change), the practitioner (e.g., characteristics and skills), the relationship between the practitioner and the client (e.g., alliance), expectations of FMP (e.g., hope because of being in treatment), extra‐therapeutic factors (e.g., characteristics of life or environment of the client that impacts change, such as social support) and non‐specific treatment characteristics (e.g., variables found in many treatments such as reframing or behavioral regulation) (Lambert & Barley, 2001; Sprenkle & Blow, 2004). In addition to these common factors, specific factors contribute to the effectiveness of care such as the contents of an intervention and the structure within which the contents are provided (Wampold, 2013), also denoted as practice and program elements, respectively.

Practice and program elements of interventions may be important determinants of the effectiveness of care for FMP. For example, practice and program elements can only be effectively provided when they are combined with adequate common factors, that is when the practitioner has a good relationship with the client or the client is engaged in care (Harder et al., 2020). Moreover, the practice and program elements that constitute an intervention can contribute to the effects of common factors (Carr, 2009; Sprenkle & Blow, 2004). In other words, when the elements that are provided within the intervention are effective, this may contribute to engagement of the client and to the quality of the relationship between client and professional. Some studies even show that the practice and program elements of a specific intervention may exceed the effects of common factors, especially in case of more complex problems (Stevens et al., 2000). Unfortunately, evidence still lacks on the practice and program elements that are essential for positive outcomes in case of FMP.

Current studies focusing on effective practice and program elements of interventions in child and youth care mainly focus on specific problems of FMP, such as behavioral problems of the child (Garland et al., 2008; Leijten et al., 2019; van der Pol et al., 2019; Wyatt Kaminski et al., 2008), child mental problems (Chorpita & Daleiden, 2009; Lindsey et al., 2014), imminent out‐of‐home placement (Lee et al., 2014), or child maltreatment (Gubbels et al., 2019, 2021; van der Put et al., 2018). Some of these studies focus on practice and program elements that are common in effective interventions, but it remains unknown to which degree an element relates to a specific outcome (Chorpita & Daleiden, 2009; Garland et al., 2008; Lee et al., 2014; Lindsey et al., 2014; van der Pol et al., 2019). Other studies determined elements that are associated with positive outcomes, but only for some, specific, problems of FMP (Gubbels et al., 2019, 2021; Leijten et al., 2019; van der Put et al., 2018; Wyatt Kaminski et al., 2008). Consequently, the full range of elements provided within interventions for FMP is not covered within these studies. Given the wide range and complexity of problems of FMP, the available evidence does not allow decisive conclusions about effective elements in care for FMP.

Due to the wide range and complexity of problems in these families, the effectiveness of practice and program elements may greatly vary across their subgroups. This mix of problems may include behavioral problems of the child, parenting problems, and social network problems. Moreover, some families are coping with comorbidities such as intellectual disabilities (Bodden & Deković, 2016), psychiatric problems (Bodden & Deković, 2016), and substance use (Asen, 2007; Bodden & Deković, 2016) of the child and/or the parent. The effects of interventions vary across these subgroups, probably also depending on the degree to which the intervention can be tailored to the needs of each subgroup (Austin et al., 2005; Wade et al., 2008). Knowledge on which practice and program elements are most effective for the subgroups within FMP may therefore be helpful in tailoring interventions to their specific needs.

The taxonomy of interventions for FMP (TIFMP) provides an excellent opportunity to assess how elements of interventions affect FMP and FMP subgroups (Visscher et al., 2018). The TIFMP enables to systematically register the practice and program elements of a wide range of interventions for FMP. It consists of 53 practice elements divided into 8 main categories (e.g., assessment of problems, working on change, and activating the social network) and eight program elements (e.g., number of visits, duration of visits, intervision, or supervision for the practitioner). In a previous study, we found that the TIFMP can reliably measure the content and structure of these interventions as reported in their intervention manuals (Visscher et al., 2018). Furthermore, the TIFMP makes it possible to assess which elements in interventions for FMP are similar, and which elements are part of the interventions in daily practice (Visscher et al., 2020a, 2020b). Evidence on the effectiveness of these elements may enhance selection of the best interventions for FMP or subgroups of FMP. The aim of this study is thus to assess the degree to which practice and program elements contribute to the effectiveness of interventions for FMP and FMP subgroups.

## METHODS

### Study design

We performed a quasi‐experimental study, adjusting for differences in prognosis between FMP's by using propensity scores (PS) or quasi‐randomization. We collected data, using questionnaires, from January 2017 till April 2019. Questionnaires were filled in by practitioners (child and youth care social workers, family coaches, and/or therapists) at the beginning (T0) and the end of the intervention (T1). In addition, every 4 weeks during the intervention period, practitioners registered the elements which they provided to families participating in the study. Caregivers who received the intervention gave informed consent and filled in questionnaires at T0, T1, and 3 months after conclusion of the intervention (T2).

### Study setting and participants

To obtain a sample of FMPs, we used a three‐step procedure. First, of interventions targeting FMP we selected eight with at least a moderate effect size of 0.5 in the Dutch context regarding core outcomes, like problem behavior of the child or parenting stress. The eight interventions were Multisystemic Therapy (MST), Multidimensional Family Therapy (MDFT), Intensive Family Treatment (IFT), Families First (FF), Family Central (FC), Parent Management Training Oregon (PMTO), 10 for the Future (10ftF), and Triple P 4–5 (Evenboer et al., 2018). These interventions aim at families with severe parenting problems (MST, MDFT, PMTO, and Triple P 4/5) and families with multiple and complex problems in different life domains like severe parenting, and socio‐economic and mental health problems (IFT, FF, FC, and 10ftF). More information on these interventions can be found elsewhere (Visscher et al., 2020b).

Second, we approached 47 child and adolescent social care organizations in the Netherlands which provided at least one of the eight selected interventions. Of these, 26 were willing to participate in this study and provided teams of practitioners. Organizations that did not want to participate indicated either that they had already taken part in another study or did not wish to spend scarce manpower and resources to participate in a study. Nonparticipating and participating organizations did not differ in size or target population.

Third, we included FMP that had received one of the selected interventions between January 2017 and April 2018 (for FC and 10ftF), and families that received the other interventions between January 2017 and July 2018. Eligible participants were caregivers who were able to complete questionnaires in Dutch, and who had a child of 4 years or older who was targeted for the intervention. Children below 4 years of age were excluded in our study because some of the interventions included in this study are not suitable for children of this age (e.g., MST or MDFT) (Visscher et al., 2018). In addition, one of the questionnaires to measure care use of FMP (not used in this study) can only be filled in by parents of children above 4 years of age, because it was developed for economic evaluations of systemic interventions for children with severe behavioral problems above 4 years of age. We excluded 12 families because the registered child was younger than 4 years old at the start of the intervention. We also excluded families receiving Triple P 4–5 and FF because these families were too few (one and nine families, respectively) to be analyzed, and the interventions were not long enough to allow for measurement of change.

### Treatment conditions

We measured provided practice and program elements by using the TIFMP. Regarding practice elements, we looked for those frequently provided in combination.

#### Identification of provided practice and program elements

To identify elements provided to FMPs, practitioners filled in the TIFMP, which is a taxonomy to systematically measure practice elements (distinct techniques used by practitioners to promote positive outcomes) and program elements (aspects of the intervention design or service delivery system) (Visscher et al., 2018). For this study, we developed an online version of the TIFMP. Practitioners received a link to fill it in every 4 weeks during the entire intervention, for each of their cases separately. Practitioners had 10 days to fill in the questionnaire and received a reminder after 5 days. Practice elements consisted of 53 elements, divided into the following eight main categories:

- *Assessment of problems*: practice elements aimed at collecting and categorizing information about the family and the problems they experience (e.g., analysis of competencies);
- *Planning and evaluation*: practice elements aimed at translating family problems into goals and/or at evaluating these goals (e.g., designing the treatment plan);
- *Working on change*: practice elements aimed at realizing change (e.g., working on communication and interaction);
- *Learning parenting skills*: practice elements aimed at improving parenting skills (e.g., learning to set rules);
- *Helping with concrete needs*: practice elements aimed at easing the burden of practical everyday challenges (e.g., helping with financial tasks);
- *Activating the social network*: practice elements aimed at engaging the family's social network to help and support the family (e.g., mobilizing and expanding the social network);
- *Activating the professional network*: practice elements aimed at enhancing goals, appointments, and procedures involving other practitioners who work with the family (e.g., coordinating the approach with other professionals and/or organizations);
- *Maintaining practitioner*‐*client collaboration*: practice elements aimed at maintaining and promoting practitioner‐client collaboration (e.g., talking about expectations).

Regarding each of the 53 practice elements, we asked practitioners whether they had provided that element in the past 4 weeks (yes/no), and if yes, to indicate with what frequency (i.e., in less than half of the visits, half or more of the visits, or all visits during the past 4 weeks).

Provided program elements involved registration of the number and mean duration of visits to the family in the past four weeks, whether the practitioner had telephone contact with the family between visits in the past 4 weeks, and whether the practitioner received intervision, supervision, and/or consultation about the family in the past 4 weeks. Intervision involves discussing the family with colleagues during an organized meeting; supervision refers to discussing the family with a supervisor during an organized meeting; and consultation means discussing the family with an independent expert during an organized meeting. In order to analyze program elements, we calculated means for the number and duration of visits. This resulted in a mean number and mean duration of visits during the entire intervention. For telephone contacts, intervision, supervision, and consultation, we counted the total number of registered occurrences. This resulted in a score indicating the total number of times the practitioners had phone contacts with the family or received intervision, supervision or consultation during the entire intervention. More detailed information on the TIFMP can be found elsewhere (Visscher et al., 2018).

#### Identification of combinations of practice elements (profiles)

To identify groups of families receiving a similar combination of practice elements, we performed a Latent Profile Analysis (LPA) based on relative scores of the 53 provided practice elements. This LPA made it possible for us to analyze combinations of the elements provided; this was our aim, as practice elements are not provided to FMP in isolation (Lee et al., 2014). We calculated relative scores, based on the elements provided and the intensity with which they were provided. We categorized provision per element as 0 (not provided), 1 (provided < half of visits), 2 (provided half or more of visits), or 3 (always provided). Next, for each family, we summed scores across questionnaires filled in by the practitioner and divided this sum by the maximum scores (the number of questionnaires times 3), resulting in relative scores per practice element, from 0 (small share of element in the overall intervention) to 1 (large share of element in the overall intervention).

In the LPA, we estimated models with one to four profiles and selected the best model based on the Bayesian information criterion (BIC), the Lo–Mendell–Rubin likelihood ratio test (LMR‐LRT), the bootstrap likelihood ratio test (BLRT), mean posterior probabilities, and interpretation of the resulting profiles. Entropy reflects profile distinctiveness, where values close to 1 indicate high quality of profile assignment (Berlin et al., 2014). BIC values decreased, that is, improved, with increasing number of profiles (as listed in Table S1), but the LMR‐LRT test for the four‐profile model was nonsignificant (*p* = .17), indicating that the four‐profile model did not fit significantly better than the three‐profile model. The BLRT showed that the four‐profile solution fitted significantly better than the three‐profile solution. However, this indicator often keeps improving with the addition of profiles and a graphical examination of “elbow plots” is recommended to decide on the best, most parsimonious, solution (Petras & Masyn, 2010). Indeed, the BIC elbow plot showed an inflection point in the curve, suggesting that the optimal number of profiles was reached at the three‐profile solution. Furthermore, the four‐profile solution included one relatively small group (7.0%), and profiles were more difficult to interpret (this solution consisted of a low, middle‐low, middle‐high and high intensity profile). Therefore, we chose the three‐profile solution. Mean posterior probabilities of this three‐profile solution ranged from 0.970 to 0.993, and the entropy was 0.95, “very high” (Berlin et al., 2014).

The three identified practice element profiles represent three profiles of care provided to FMP, differing in focus and intensity: a relatively more explorative/supportive profile, a combined explorative/supportive and action‐oriented profile, and a relatively more action‐oriented profile. These profiles are further described in Box 1, Figure S1, and Table S2.

Box 1 **Description of identified practice elements profiles**

The three identified practice element profiles represent three care trajectories that differ in the intensity in which practice elements are provided and the focus of care. The following three profiles were identified:

Profile 1. Relatively more supportive and explorative profile (received by 38.4% of FMP)

*Intensity*: This profile consists of elements with a low relative score, meaning that they had a small share in the trajectory. These can be trajectories of longer duration and focused on a wide range of problems (broad focus), resulting in lower relative scores of practice elements.

*Interventions*: IFT and 10ftF are overrepresented in this profile, whereas MST and MDFT are underrepresented.

*Focus*: Elements provided within profile 1 are focused relatively more on offering support (emotional support and quality of relationship), exploration (discussing the guiding question and expectations), and creating positive relationships within the family (communication, collaboration, desired behavior, and applying reinforcements). This focus characterizes a relatively more explorative/supportive profile.

*Practice elements that had the highest share in this profile*: offering emotional support (0.52), discussing the guiding question (0.44), working on communication and interaction (0.42), working on the quality of the relationship (0.41), and working on desired behavior (0.40).

Profile 2. Combination of supportive/explorative and action‐oriented profiles (received by 40.9% of FMP)

*Intensity*: This profile consists of elements provided with medium intensity, meaning that the elements had a higher share in profile 2 compared to profile 1, but not as high as elements in profile 3.

*Interventions*: IFT was underrepresented within profile 2.

*Focus*: The focus of elements provided within profile 2 is a combination of the relatively more supportive/explorative profile and the relatively more action‐oriented profile. Compared to profile 1, greater focus was on communication, desired behavior, and regulating problem behavior. Less focus was on exploration and support (guiding question, expectation of care, and evaluating working points), although profile 2 included focus on enhancing motivation and analyzing competencies.

*Practice elements that had the highest share in this profile*: offering emotional support (0.72), working on communication and interaction (0.68), working on desired behavior (0.68), working on the quality of the relationship (0.67), and working on recognizing, avoiding and coping with situations eliciting problem behavior, and help with eliminating these causes (0.63).

Profile 3. Relatively more action‐oriented profile (received by 20.8% of FMP)

*Intensity*: This profile consists of elements with a high relative score, meaning that they had a high share in the trajectory. These trajectories may be characterized as focused trajectories, whereby care is targeted to a limited number of problems for which similar elements are provided. This may be either trajectories of short duration, in which relative scores were higher due to time pressure, or treatments of longer duration, in which elements were often provided (e.g., every visit during a 6‐month intervention).

*Interventions*: Within profile 3, MST is overrepresented and IFT is underrepresented.

*Focus*: The focus of elements provided within profile 3 is on the relationship between family members (communication, handling conflicts, and analysis of family system) and on regulating (problem) behavior (working on desired behavior, learning to set rules, and recognizing and coping with problem behavior). This focus characterizes a relatively more action‐oriented profile.

*Practice elements that had the highest share in this profile*: working on desired behavior (0.88), working on communication and interaction (0.86), working on the quality of the relationship (0.85), offering emotional support (0.83), and working on recognizing, avoiding and coping with situations eliciting problem behavior, and help with eliminating these causes (0.82).

### Quasi‐randomization: computation of PS

We used PS to adjust for differences in prognosis between treatment groups (Winship, 1992). We computed these scores from the socio‐demographic and problem‐related baseline variables, and the baseline scores on internalizing and externalizing problems, parenting stress, and social contacts, as specified hereafter. Probabilities for each family to receive each practice element profile were estimated with a multinomial logistic regression model, predicted by baseline scores on all 17 variables. Variables were included as categorical variables, except for social contacts, parenting stress, internalizing problems, and externalizing problems. These four variables were included as continuous variables (e.g., *T*‐scores). Missing values were imputed with arbitrary but constant values, and a missingness indicator for each variable was included in the PS estimation, coded 1 if there was a missing value for the variable and 0 if not (Cham & West, 2016; D’agostino et al., 2001; Haviland et al., 2007; Rosenbaum, 2010).

### Procedure

First, during an introductory meeting, we informed participating teams of practitioners about the study (background and aims), the procedures, and the questionnaires used. In this meeting, we also trained them in how to register provided elements using the TIFMP. During the first or second home visit, the practitioners provided the caregivers with oral or written information, by means of a leaflet or a video, on the aims and the procedure of the study. We next obtained signed informed consent from all participants.

We collected data using a web‐based questionnaire system (BergOp). Directly after obtaining informed consent, we sent the T0 questionnaire to the practitioner and the caregiver. We sent the T1 questionnaire on the registered end date of the intervention, and we sent the T2 questionnaire 3 months later. For all above‐mentioned questionnaires, respondents had 21 days to fill in the questionnaire and received a reminder after 14 days. After every completed questionnaire, caregivers were rewarded with a gift token of € 10.

### Measures

#### Primary outcome

The primary outcome of the study pertained to the child's internalizing and externalizing problems, measured by means of the Child Behavior Checklist (CBCL) and reported by the caregiver (Achenbach & Rescorla, 2001). This is a widely used age‐normed measure with good reliability and validity (Nakamura et al., 2009). We used the 35‐item externalizing broad‐band scale (Achenbach & Rescorla, 2001) to assess children's levels of externalizing problems and the 32‐item internalizing broad‐band scale of the CBCL (Achenbach & Rescorla, 2001) to assess children's levels of internalizing problems. All items in the CBCL were rated on a three‐point Likert‐type scale: (0 = not true, 1 = somewhat true, and 2 = certainly true). We computed *T*‐scores, indicating the deviation of the score from the mean of the norm population, and used these for analyses. Higher *T*‐scores indicate that adolescents were believed by caregivers to experience more problems. The Cronbach's alphas of the study sample for CBCL internalizing and externalizing problems were 0.88 and 0.92, respectively.

#### Secondary outcomes

Secondary outcomes were parenting stress and the family's social contacts. We measured caregiver‐reported parenting stress at T0, T1, and T2 using the Parenting Stress Questionnaire (Opvoedingsbelasting Vragenlijst) (Vermulst et al., 2012). This questionnaire consists of 34 items with a four‐point Likert scale (0 = not true, 1 = somewhat true, and 2 = certainly true). We summed scores on all items to compute a *T*‐score for total parenting stress, indicating the score's deviation from the mean of the norm population. A higher *T*‐score indicates a higher level of parenting stress; the Cronbach's alpha of the study sample was 0.94.

We measured social contacts using the Social Contacts subscale of the Questionnaire Family Functioning of Parents (VGFO, Vragenlijst Gezinsfunctioneren van Ouders) as reported by the caregiver (Veerman et al., 2012). This outcome measure was chosen because positive social contacts contribute to the well‐being of FMP and social isolation is a real risk for these families (Sousa et al., 2007). Consequently, an important aim of interventions for FMP is to strengthen this network. The VGFO consists of five items with a four‐point Likert scale: (1 = “does not apply to our family or to me”, 2 = “applies somewhat to our family or to me”, 3 = “applies accurately to our family or to me”, 4 = “applies entirely to our family or to me”). We added scores on all five items to compute a *T*‐score for social network problems, indicating the score's deviation from the mean of the norm population. A higher *T*‐score indicates more social contacts. The Cronbach's alpha was 0.79.

#### Background characteristics

To be able to correct for initial differences between FMPs, we obtained data at baseline on an extensive set of socio‐demographic characteristics and problem‐related characteristics. Socio‐demographic characteristics pertained to age (4–12 vs. 12+, i.e., adolescent) (Perry et al., 2011; van der Stouwe et al., 2014) and gender of the child (van der Stouwe et al., 2014), and ethnicity of the caregiver (non‐industrialized/industrialized [i.e., born in Europe, excluding Turkey], North America, Oceania, Indonesia, and Japan) (CBS, 2020). Educational level of the mother was classified into: “low” (none to maximum lower general secondary education), “medium” (intermediate vocational education or apprenticeship to pre‐university secondary education), and “high” (higher vocational education or university) (Kleefman et al., 2014). Marital status (Al et al., 2012) was dichotomized into “one‐parent families” (divorced/not living together, widowed, and single) or “two‐parent families” (married or living together with partner). Problem‐related variables included financial problems (having problems managing the household income in the past year: No [original answers: “No, not at all” and “No, but I have to keep expenses low”] and Yes [original answers: “Yes, a little” and “Yes, a lot”]). We measured comorbid disorders by asking practitioners whether they suspected intellectual disabilities (Perry et al., 2011), psychiatric problems, or substance use (Henderson et al., 2010) in the caregiver, child, or both caregivers and child (“Yes”, “No”, or “I don't know”); the last answer was considered as missing (*n* = 27 in intellectual disability, *n* = 112 in psychiatric problems, and *n* = 35 in substance use). To measure other care involved in the family, we asked the practitioner whether such care was involved (yes or no).

### Statistical analyses

We first assessed the background characteristics of the sample. Next, we assessed the effects of practice element profiles and program elements on improvement in the outcomes, both short (i.e., T0 to T1) and longer term (i.e., T0 to T2). We used path models to assess these relationships. In these models, the dependent variables were social contacts, internalizing and externalizing problems, and parenting stress, assessed at the end and 3 months after conclusion of the intervention. For the analyses on the effectiveness of practice element profiles, the independent variables were the practice element profiles (using the explorative/supportive profile as reference category), the baseline score on the independent variable, and the PS. For the analyses on the effectiveness of program elements, the independent variables were the program element, the baseline score on the independent variables, and the PS. We analyzed this separately for short‐ and longer‐term outcomes. Third, we used multiple group chi‐square difference analyses to assess whether the effects of practice element profiles and program elements differed per subgroup: families with and without caregivers, or children with intellectual disabilities, psychiatric problems, and substance use. We performed analyses in Mplus (Muthén & Muthén, 2012). To make maximum use of the available data, we used the full information maximum likelihood (FIML) estimation procedure as implemented in Mplus to deal with the missing values (Muthén & Muthén, 2012).

## RESULTS

We first describe the characteristics of the sample and then the effects of the practice element profiles and program elements for all FMP and for FMP subgroups.

### Characteristics of sample

We included a total of 499 families, 26 of which were excluded due to missing data on elements provided. This resulted in a total sample of 473 families; of these, 234 received IFT, 59 MDFT, 130 MST, 33 PMTO, 11 10FtF, and 6 FC. Table 1 shows the baseline characteristics of families for the three practice element profiles. Families in the three profiles differed significantly with regard to the child's age, child's intellectual disabilities, child's substance use, ethnicity of the caregiver, and perceived parenting stress. In addition, Table 1 shows that although belonging to a particular profile is not arbitrary, nevertheless FMP with similar problems do not always receive the same practice element profile.

**TABLE 1:** Baseline characteristics of families with multiple problems that received the explorative/supportive, combined, and/or action‐oriented profiles of care

	Explorative/supportive (*n* = 180)	Combined (*n* = 195)	Action‐oriented (*n* = 98)	*p* Value^a^
Gender of the child				
Boy	117 (65.0%)	111 (56.9%)	56 (57.1%)	.226
Age of child, mean (*SD*)	10.6 (3.9)	12.5 (3.8)	13.4 (3.4)	<.001
Intellectual disability child				
Yes	61 (35.9%)	39 (23.1%)	14 (17.7%)	.003
Intellectual disability parent				
Yes	35 (20.6%)	25 (14.8%)	11 (13.9%)	.264
Psychiatric problems child				
Yes	66 (48.9%)	66 (48.9%)	29 (43.9%)	.771
Psychiatric problems parent				
Yes	64 (47.4%)	68 (50.4%)	35 (53.0%)	.740
Substance use child				
Yes	4 (2.4%)	22 (13.2%)	11 (14.1%)	.001
Substance use parent				
Yes	13 (7.9%)	12 (7.2%)	7 (9.0%)	.888
Other care involved in family				
Yes	100 (57.5%)	91 (49.7%)	45 (51.1%)	.316
Ethnicity parent				
Industrialized	120 (96.8%)	121 (96.8%)	54 (87.1%)	.008
Marital status parent				
Two‐parent family	82 (63.6%)	89 (62.7%)	34 (53.1%)	.334
Educational level parent				
Low	30 (26.1%)	24 (19.5%)	13 (26.5%)	.701
Middle	66 (57.4%)	82 (66.7%)	27 (55.1%)	
High	19 (16.5%)	17 (13.8%)	9 (18.4%)	
Financial problems parent				
Yes	47 (36.7%)	43 (31.9%)	20 (32.3%)	.677
Internalizing problems, mean (*SD*)	63.6 (10.3)	62.6 (9.6)	63.9 (10.1)	.654
Externalizing problems, mean (*SD*)	64.6 (10.3)	66.3 (10.5)	67.8 (8.7)	.155
Parenting stress, mean (*SD*)	64.9 (11.8)	68.7 (9.5)	68.9 (9.8)	.003
Social contacts, mean (*SD*)	43.3 (12.8)	41.9 (11.8)	42.8 (11.0)	.589

Note

Reported percentages are valid percentages. Differences in background characteristics across practice elements profiles were assessed by means of chi‐square tests (for categorical baseline variables) or ANOVA (for continuous baseline variables).

^a^ Results of chi‐square and ANOVA.

### Effects of practice and program elements for FMP

Regression analyses revealed no significant differences between practice element profiles regarding changes in parenting stress, internalizing, and externalizing problems, either short or longer term (Table 2). In contrast, in several outcomes we found that the intensity of the provided program elements, particularly telephone contacts and intervision, had significant effects on changes. Having more frequent telephone contacts between visits was associated with a greater increase in social contacts in the short term, a greater decrease in parenting stress (short and longer term), and a greater decrease in internalizing problems (in the longer term). The practitioner's receiving of more intervision was associated with a greater short‐term decrease in the family's internalizing and externalizing problems. We further found that more supervision was associated with a smaller short‐term increase in social contacts, and more frequent consultation was associated with a smaller longer‐term increase in social contacts. Finally, more frequent visits were associated with a smaller longer‐term increase in social contacts.

**TABLE 2:** Effects of practice elements profiles and program elements on intervention outcomes: Standardized regression coefficients, for short (T1) and longer term (T2)

	Social contacts^a^		Parenting stress^a^		Internalizing problems^a^		Externalizing problems^a^	
	T1	T2	T1	T2	T1	T2	T1	T2
	*β*	*β*	*β*	*β*	*β*	*β*	*β*	*β*
*Practice elements profiles*								
Explorative/supportive								
Combined	.062	−.064	.065	−.019	−.017	.031	−.087	.077
Action oriented	−.002	−.066	.075	.043	.003	.033	−.002	.093
*Program elements*								
Number of visits	−.013	−.177**	.075	.071	.004	.057	.057	.057
Duration of visits	−.075	−.074	.062	.099	.019	.129	.015	.054
Telephone contacts	.145*	.146	−.113*	−.131**	−.027	−.188*	−.008	−.140
Intervision	.031	.091	−.072	.016	−.183*	−.042	−.123*	−.028
Supervision	−.158*	−.147	.032	−.051	−.062	−.004	−.088	−.018
Consultation	−.006	−.137*	−.047	−.007	.052	.069	−.020	.004

Note

In these analyses we controlled for the propensity score and baseline scores on social contacts, parenting stress, internalizing, and externalizing problems

Profile 1 (explorative/supportive profile) was regarded as the reference group.

*p < .05, **p < .01, ***p < .001.

^a^ To make maximum use of the available data, we used the full information maximum likelihood (FIML) estimation procedure as implemented in Mplus to deal with missing data on the four outcome measures. Original sample sizes for social contacts were T0 = 349, T1 = 224, and T2 = 140; for parenting stress T0 = 382, T1 = 252, and T2 = 142; and for both internalizing and externalizing problems T0 = 276, T1 = 196, and T2 = 91.

### Effects of practice and program elements for FMP subgroups

We assessed whether the effects of practice element profiles and program elements varied across subgroups: families with and without parental and/or child psychiatric problems, families with intellectual disabilities, or families with substance use. In 144 families, the child was reported to have an intellectual disability, whereas in 304 families, the child had no intellectual disability. In 161 families, the child had psychiatric problems, and in 175 families, the child had no psychiatric problems. In 167 families, parental psychiatric problems were reported, whereas in 169 families, no parental psychiatric problems were reported. Based on power calculations regarding comparison of two regression lines by Shieh (2018) and assuming small to moderate differences between subgroups regarding the effects of practice and program elements, we conclude that these subsample sizes are sufficient for realizing a power of at least 0.80. For the subgroups child and parental substance use, and parental intellectual disabilities, we could not assess the short‐ and longer‐term effects of practice element profiles and the short‐term effects of program elements. In addition, we could not assess the longer‐term effects of program elements on the subgroup regarding child intellectual disabilities. This was due to an inadequate number of FMP with these characteristics in the analyses. A complete overview of these results can be found in Table S3.

Regarding practice elements, we found that the effect of the “combined explorative/supportive and action‐oriented” profile varied for families with and without parental psychiatric problems. In case of parental psychiatric problems, this combined profile was associated with a greater increase in social contacts in the short term, whereas in families without these problems no difference was found with the reference category (i.e., the explorative/supportive profile) (Δχ^2^(1) = 6.148, *p* = .0132). In the longer term, this combined profile was associated with a smaller increase in social contacts in families without parental psychiatric problems, whereas in families with these problems, no difference was found with the reference category (Δχ^2^(1) = 4.171, *p* = .0411).

Regarding program elements, the effects varied for three subgroups. First, the effect of program elements varied for families with or without a child with an intellectual disability. Exclusively in families with a child with intellectual disabilities, a longer duration of visits was associated with a smaller short‐term increase in social contacts (Δχ^2^(1) = 4.535, *p* = .0332). Second, the effect of telephone contacts varied for families with and without a child with psychiatric problems. When the child had psychiatric problems, more telephone contacts were associated with a greater reduction in parenting stress, both short term (Δχ^2^(1) = 5.851, *p* = .0156) and longer term (Δχ^2^(1) = 9.268, *p* = .0023); in families where the child had no psychiatric problems, this was not the case. In addition, in families of a child with psychiatric problems, more intervision was associated with a greater longer‐term decrease in internalizing problems of the child; this was not the case in families without a child's psychiatric problems (Δχ^2^(1) = 7.995, *p* = .0047). In case of child psychiatric problems, more frequent consultation was also associated with a greater longer‐term decrease in internalizing problems of the child; this was not the case in families without child psychiatric problems (Δχ^2^(1) = 5.481, *p* = .0192). Finally, when a child had psychiatric problems, more supervision was associated with a smaller longer‐term increase in social contacts; in families without child psychiatric problems, this was not the case (Δχ^2^(1) = 7.448, *p* = .0064).

Third, the effects of program elements varied for families with and without parental psychiatric problems. If the parent had psychiatric problems, more intervision was associated with a greater short‐term increase in social contacts, whereas in the subgroup where parents had no psychiatric problems, more intervision was associated with a smaller increase in social contacts (Δχ^2^(1) = 11.171, *p* = .0008). Furthermore, if a parent had psychiatric problems, more supervision was associated with a smaller longer‐term decrease in child internalizing and externalizing problems. In families where no parent had psychiatric problems, more supervision was associated with a greater decrease in externalizing problems (Δχ^2^(1) = 7.811, *p* = .0052) but not with changes in internalizing problems (Δχ^2^(1) = 4.427, *p* = .0354). Finally, in families without parental psychiatric problems, a longer duration of visits was associated with a smaller longer‐term increase in social contacts, whereas in families with parental psychiatric problems, a longer duration was not associated with changes in social contacts (Δχ^2^(1) = 4.720, *p* = .0298).

## DISCUSSION

We assessed the degree to which practice and program elements contribute to the effectiveness of interventions for FMP and FMP subgroups. First, we found that differences in the content of care (practice elements) for FMP did not influence the effectiveness of care. Second, however, we found some specific differences in the structure of care (program elements) for FMP that did lead to differences in effectiveness. Especially in cases of more frequent telephone contacts between visits and more frequent intervision for the practitioner, effects were greater for some outcome measures. Further, we found that the effectiveness of practice elements varied for caregivers with or without psychiatric problems. FMPs with parental psychiatric problems showed greater improvements when they received the “combined explorative/supportive and action‐oriented profile”. The effect of some specific program elements varied for subgroups with and without child and parental psychiatric problems and child intellectual disabilities. Frequent telephone contacts and intervision led to greater improvements in cases of child or parental psychiatric problems, and of intellectual disabilities of the child, whereas more supervision and a longer duration of visits were related to smaller improvements in cases of psychiatric problems.

We found that differences in the content of care for FMP (i.e., intensity and focus) did not affect changes in psychosocial problems, social contacts, and parenting stress. Families receiving the explorative/supportive profile may thus experience the same treatment effects when receiving the combined or action‐oriented profile, and vice versa. This finding confirms the conclusions of Gubbels et al., (2019) in their meta‐analysis of the effectiveness of elements of parenting programs designed to prevent and reduce child maltreatment; these authors also found most elements to be equally effective. An explanation for our finding of an equal effectiveness of the content of FMP interventions might be that variation in effectiveness was diminished by including only effective interventions (ES > 0.5). Probably related, the content of these eight interventions was found to be quite similar (Visscher et al., 2020b). This overlap may explain the lack of significant differences found in the effectiveness of combinations of elements provided within these interventions.

Our finding that differences in the content of interventions for FMP did not affect their outcomes does, however, not indicate that this content does not contribute to the effect of the intervention. This content may still add to effectiveness, if compared to not receiving care at all. However, the lack of variance in the content of the effective interventions and thus in the practice elements that were provided to FMP hindered the identification of (combinations of) practice elements that contribute to positive outcomes for FMP. A next step to improve the effectiveness of care for FMP would therefore be to disentangle the specific elements within the identified practice element profiles that make this care more effective overall. In addition, not only the elements provided and their intensity but also the quality of provision and the order in which the elements are provided might influence their effects and should therefore be addressed in future research on effective elements of interventions for FMP.

Second, we found some differences in the structure of care for FMP, particularly the degree of telephone contacts that affected the effectiveness of care for some outcome measures. Especially families with child and parental psychiatric problems seemed to benefit from these contacts. More frequent telephone contacts with families might indicate more intensive trajectories of monitoring on the part of practitioners. FMP were found to benefit from such regular contacts (Holwerda et al., 2014), having stayed connected with their practitioner between visits, received feedback on homework, made new appointments, and been regularly motivated. These benefits may be more clear in families with psychiatric problems, because they meet needs related to deficits in parenting control behaviors (Johnston et al., 2012) as well as higher levels of stress (Johnston & Mash, 2001). A study on chronic problems of FMP also reported that approaches that emphasize monitoring may be more suitable for these families (Chaffin et al., 2011). A greater intensity of telephone contacts to support and monitor the family between visits therefore seems valuable for reaching more positive outcomes in FMP.

Third, we found more frequent intervision for the practitioner to be beneficial for some outcomes in FMP, reflecting the importance of mutual exchange between practitioners. Effects of intervision were even greater in cases of child and parental psychiatric problems. Although scarce, the research on the effectiveness of intervision suggests that intervision is important for professional development (Golia & McGovern, 2015) and for effective provision of the intervention (Holwerda et al., 2014). Intervision allows practitioners to interact with each other, providing mutual support, as well as shared experience and advice, on how to cope with difficult cases and specific problems (Golia & McGovern, 2015). An important difference with supervision is that intervision takes place between colleagues with similar clinical expertise, whereas supervision is often considered to be more hierarchical (Golia & McGovern, 2015). Intervision is thus a more accessible way to obtain emotional and practical support of colleagues and share experiences, tips, and tricks directly related to a specific problem that is raised by a practitioner. Discussing experiences with colleagues familiar with the context in which care is delivered may help practitioners to better match care to the complex and chronic problems of FMP.

Fourth, we had some puzzling findings for improvements in social contacts of FMP and FMP subgroups regarding the “combined explorative/supportive and action‐oriented” practice element profile and some program elements. Several explanations may apply. First, in the short term, the combined profile seemed more effective than the explorative/supportive profile in improving social contacts of families with parental psychiatric problems. However, in the longer term, this profile was associated with fewer improvements in social contacts of families without psychiatric problems. It is unclear which elements of this profile led to these outcomes, in particular because elements regarding the social network are underrepresented in care for FMP, both in this study and in general (Visscher et al., 2020a). Second, we found more frequent visits, supervision, and consultation to be generally associated with less improvement in social contacts of FMP. Some program elements, such as supervision and longer duration of visits, also had less effect in subgroups with child and/or parental psychiatric problems. This suggests that these program elements are less effective in subgroups with more complex or chronic problems. We controlled for problems of FMP at baseline, but problems may become even more apparent or severe during care, requiring practitioners to request more supervision or consultation, and to visit the family more often. This requires further study.

### Strengths and limitations

Important strengths of this study are that we collected detailed data on provided practice and program elements in a “real world” setting and considered differences between families at baseline by adjusting for PS. This allowed us to make more valid inferences of effectiveness (Rosenbaum & Rubin, 1983), thus accounting for the complexity of problems faced by FMPs. Another strength of our study is its longitudinal design, with a follow‐up measurement 3 months after conclusion of the intervention.

Our study also has some limitations. First, some practitioners may have interpreted elements differently, leading to overreporting or underreporting. However, we trained practitioners in filling in the TIFMP and provided additional descriptions of elements in the questionnaire, which was also made in collaboration with practitioners. Second, our use of 4‐week time periods, within which practitioners had to register the elements as provided, may have led to problems of recall. However, practitioners indicated that they could do so validly, largely reducing the likelihood of bias. Finally, we could not randomize families. We therefore used PS to control for differences in prognosis between FMPs. Although we used a comprehensive set of variables to check for differences in the prognosis of FMP, the prognosis of some groups may have differed slightly.

### Implications

Our findings may have implications for researchers and practitioners who are engaged in care for FMP. First, we found no major differences in the effectiveness of different contents of interventions, suggesting that these are equally effective. It might therefore be more efficient to strengthen the content of a small number of interventions, rather than developing and investing in further interventions with similar contents. The findings of our study do, however, not allow to advice changes in the content of interventions for FMP, because no specific (combinations of) practice elements were found that made care for FMP more effective. To strengthen existing interventions, further research on the elements most effective for FMP is crucial, to indicate which should be added or omitted.

Second, more frequent telephone contacts between families and practitioners, as well as intervision for the practitioner, enhance the effectiveness of care for FMP and for specific subgroups, underlining the value of such contacts for all FMP interventions. Frequent phone contacts between visits to the family should thus be routine care and this similarly holds for frequent intervision meetings for all practitioners working with FMP. The latter holds even more in case of psychiatric problems of the child or parent. Therefore, as the current nature and frequency of intervision meetings varies widely across interventions (Visscher et al., 2020b), more frequent and regular scheduling of intervision might help the practitioner to cope more successfully with the complex and quickly shifting problems of FMP, thereby improving care for these families. In families in which the child has psychiatric problems, the number of phone contacts should be high as this was found to reduce parenting stress. Other forms of digital communication could also be helpful to increase this frequency of contacts, in addition to telephone contacts.

Third, our findings that the contents of care are similar in effectiveness, but that the structures in which these contents are provided differ in effectiveness, are an important step toward tailored care for FMP. Future research should focus on further improving the contents (practice elements) of interventions by adding or omitting certain (combinations of) elements. Further analysis of the interaction between provided elements and characteristics of families, and of the most effective ways to provide elements (e.g., psycho‐education, instruction), would augment our understanding of which elements provide positive outcomes and help to strengthen and tailor interventions to specific FMPs.

## CONCLUSION

Our study showed that variations in the content of care for FMP do not affect its effectiveness, but that variations in the structure in which care is provided do affect effectiveness. Moreover, effectiveness varied for subgroups of FMP, indicating the importance of tailoring interventions to specific characteristics of FMP. This provides new routes to improve this care.

## ETHICAL APPROVAL

The Medical Ethics Committee of the University Medical Center Groningen in the Netherlands provided a waiver for this study (reference number METc2016.005, dated 7 March 7, 2016). The study was registered in the International Standard Randomized Controlled Trial Number Register (ISRCTN No. 22942273).

## Supporting information

Figure S1

Table S1

Table S2

Table S3
