# Supplementary material for: Toward tailored care for families with multiple problems: A quasi‐experimental study on effective elements of care
Source: Fam Process. 2021 Dec 21;61(2):571–90. doi: 10.1111/famp.12745 (PMC9305733; doi:10.1111/famp.12745)
Supplement: Supplementary file 1 — Figure S1 [file FAMP-61-571-s004.docx]

Supplemental Figure 1. Description of three identified profiles of care (practice element profiles): their focus and the intensity in which elements were provided
