# Supplementary material for: Toward tailored care for families with multiple problems: A quasi‐experimental study on effective elements of care
Source: Fam Process. 2021 Dec 21;61(2):571–90. doi: 10.1111/famp.12745 (PMC9305733; doi:10.1111/famp.12745)
Supplement: Supplementary file 2 — Table S1 [file FAMP-61-571-s002.docx]

Supplemental Table 1. *Fit Indices of models with 1-4 profiles of care (n = 473)*

| Number of profiles | BIC | Entropy | LMR-LRT | BLRT | Post. Prob |
| --- | --- | --- | --- | --- | --- |
| 1 | 586.361 | 1.000 |  |  |  |
| 2 | -4608.404 | 0.978 | 0.0000 | 0.0000 | 0.994-0.995 |
| **3** | **-6101.500** | **0.950** | **0.0008** | 0.0000 | 0.970-0.993 |
| 4 | -6466.581 | 0.953 | 0.1616 | 0.0000 | 0.970-0.987 |

*Note.* BIC = Bayesian Information Criterion; LMR-LRT = Lo Mendell Rubin adjusted likelihood ratio test; BLRT = Bootstrap likelihood ratio test; Post. Prob = Posterior probability of classification into the most likely profile.
