# Supplementary material for: Toward tailored care for families with multiple problems: A quasi‐experimental study on effective elements of care
Source: Fam Process. 2021 Dec 21;61(2):571–90. doi: 10.1111/famp.12745 (PMC9305733; doi:10.1111/famp.12745)
Supplement: Supplementary file 3 — Table S2 [file FAMP-61-571-s001.docx]

Supplemental Table 2. Percentage of Families for Whom the Practice Element was Registered at Least Once During the Intervention, and Relative Score of the Element (Element’s Share in the Overall Intervention, Ranging from 0 to 1)

| **Practice elements** | |  | **Profile 1**  Explorative/supportive  (N = 180, % = 38.4) | | | **Profile 2**  Combined  (N = 195, % = 40.9%) | | | **Profile 3**  Action-oriented  (N= 98, % = 20.8%) | | |
| --- | --- | --- | --- | --- | --- | --- | --- | --- | --- | --- | --- |
|  | |  | *N* | *%* | *Mean relative score* | *N* | *%* | *Mean relative score* | *N* | *%* | *Mean relative score* |
| 1. **Assessment of problems** | | | | | | | | | | | |
| A1 | Discussing the guiding question | | 174 | 97% | 0.44* | 192 | 98% | 0.60* | 97 | 99% | 0.79* |
| A2 | Analysis of competencies | | 147 | 82% | 0.25 | 173 | 89% | 0.48* | 85 | 87% | 0.71 |
| A3 | Analysis of network | | 125 | 69% | 0.15 | 163 | 84% | 0.26 | 78 | 80% | 0.56 |
| A4 | Analysis of safety | | 118 | 66% | 0.20 | 165 | 85% | 0.37 | 79 | 81% | 0.72 |
| A5 | Analysis of family system | | 160 | 89% | 0.27 | 188 | 96% | 0.51 | 89 | 91% | 0.80* |
| A6 | Analysis of leisure time | | 116 | 64% | 0.15 | 156 | 80% | 0.31 | 77 | 79% | 0.50 |
| A7 | Analysis of school functioning | | 156 | 87% | 0.20 | 169 | 87% | 0.38 | 86 | 88% | 0.63 |
| A8 | Analysis of daily routine | | 139 | 77% | 0.21 | 170 | 87% | 0.39 | 80 | 82% | 0.57 |
| A9 | Analysis of individual problems | | 147 | 82% | 0.26 | 175 | 90% | 0.44 | 84 | 86% | 0.69 |
| A10 | Using homework assignments to observe and register behavior | | 122 | 68% | 0.23 | 151 | 77% | 0.38 | 76 | 78% | 0.55 |
| A11 | Using questionnaires | | 105 | 58% | 0.09 | 142 | 72% | 0.17 | 74 | 76% | 0.30 |
| A12 | Discussing results from questionnaires | | 83 | 46% | 0.10 | 102 | 52% | 0.16 | 62 | 63% | 0.27 |
| A13 | Problem assessment | | 159 | 88% | 0.26 | 188 | 96% | 0.44 | 88 | 90% | 0.68 |
| 1. **Planning and evaluation** | | | | | | | | | | | |
| B1 | Designing treatment plan | | 153 | 85% | 0.16 | 170 | 87% | 0.25 | 80 | 82% | 0.51 |
| B2 | Designing working points or (behavioral) agreements | | 166 | 92% | 0.27 | 187 | 96% | 0.51 | 86 | 88% | 0.76 |
| B3 | Evaluating working points or (behavioral) agreements | | 169 | 94% | 0.37* | 186 | 95% | 0.61* | 88 | 90% | 0.79* |
| B4 | Evaluating treatment plan | | 150 | 83% | 0.22 | 164 | 84% | 0.33 | 81 | 83% | 0.59 |
| 1. **Working on change** | | | | | | | | | | | |
| C1 | Working on recognizing, avoiding, and coping with situations eliciting problem behavior, and help with eliminating these causes | | 172 | 96% | 0.35* | 190 | 97% | 0.63* | 89 | 91% | 0.82* |
| C2 | Working on thoughts | | 127 | 71% | 0.27 | 166 | 85% | 0.43 | 79 | 81% | 0.69 |
| C3 | Working on emotions | | 142 | 79% | 0.28 | 169 | 87% | 0.50 | 79 | 81% | 0.76 |
| C4 | Working on desired behavior | | 170 | 94% | 0.40* | 183 | 94% | 0.68* | 85 | 69% | 0.88* |
| C5 | Working on undesired behavior | | 114 | 63% | 0.24 | 166 | 85% | 0.45 | 79 | 81% | 0.75 |
| C6 | Working on communication and interaction | | 161 | 89% | 0.42* | 191 | 98% | 0.68* | 87 | 89% | 0.86* |
| C7 | Working on authority relationships | | 127 | 71% | 0.25 | 178 | 91% | 0.46 | 81 | 83% | 0.76 |
| C8 | Working on daily routine | | 107 | 59% | 0.24 | 144 | 74% | 0.38 | 76 | 78% | 0.54 |
| C9 | Working on safety | | 91 | 51% | 0.22 | 152 | 78% | 0.37 | 74 | 76% | 0.66 |
| C10 | Working on generalization | | 92 | 51% | 0.25 | 151 | 77% | 0.40 | 75 | 77% | 0.65 |
| 1. **Learning parenting skills** | | | | | | | | | | | |
| D1 | Learning to apply reinforcements and positive consequences | | 141 | 78% | 0.29* | 179 | 92% | 0.46 | 85 | 87% | 0.75 |
| D2 | Learning to apply mild punishments and negative consequences | | 108 | 60% | 0.23 | 157 | 81% | 0.37 | 81 | 83% | 0.69 |
| D3 | Learning to monitor the child | | 78 | 43% | 0.21 | 128 | 66% | 0.39 | 71 | 72% | 0.64 |
| D4 | Learning to show commitment to the child | | 101 | 56% | 0.24 | 153 | 78% | 0.40 | 73 | 74% | 0.74 |
| D5 | Learning to handle conflicts | | 125 | 69% | 0.25 | 168 | 86% | 0.49 | 81 | 83% | 0.78* |
| D6 | Learning to set rules | | 133 | 74% | 0.26 | 173 | 89% | 0.46 | 83 | 85% | 0.69 |
| D7 | Learning to be responsive | | 107 | 59% | 0.22 | 150 | 77% | 0.43 | 74 | 76% | 0.70* |
| D8 | Learning to perform social skills | | 53 | 29% | 0.14 | 111 | 57% | 0.28 | 63 | 64% | 0.60 |
| D9 | Learning to collaborate | | 128 | 71% | 0.30* | 149 | 76% | 0.44 | 77 | 79% | 0.67 |
| 1. **Helping with concrete needs** | | | | | | | | | | | |
| E1 | Self-care | | 62 | 34% | 0.19 | 88 | 45% | 0.27 | 53 | 54% | 0.38 |
| E2 | Administration and financial control | | 47 | 26% | 0.14 | 55 | 28% | 0.19 | 47 | 48% | 0.30 |
| E3 | Contact with school and/or other authorities | | 135 | 75% | 0.22 | 162 | 83% | 0.33 | 82 | 84% | 0.51 |
| E4 | Housekeeping | | 37 | 21% | 0.12 | 46 | 24% | 0.14 | 46 | 47% | 0.31 |
| 1. **Activating the social network** | | | | | | | | | | | |
| F1 | Mobilizing and expanding social support | | 91 | 51% | 0.19 | 131 | 67% | 0.24 | 67 | 68% | 0.50 |
| F2 | Maintaining the social network | | 79 | 44% | 0.19 | 119 | 61% | 0.22 | 65 | 66% | 0.52 |
| F3 | Stimulating leisure time | | 106 | 59% | 0.22 | 150 | 77% | 0.31 | 71 | 72% | 0.51 |
| 1. **Activating the professional network** | | | | | | | | | | | |
| G1 | Collaborating with other professionals and/or organizations working with the family | | 163 | 91% | 0.27 | 175 | 90% | 0.35 | 83 | 85% | 0.44 |
| G2 | Coordinating the approach with other professionals and/or organizations working with the family | | 77 | 43% | 0.19 | 116 | 59% | 0.28 | 60 | 61% | 0.47 |
| G3 | Referring to other organizations or authorities | | 93 | 52% | 0.14 | 118 | 61% | 0.20 | 69 | 70% | 0.34 |
| G4 | Organizing respite care | | 17 | 9% | 0.09 | 36 | 18% | 0.17 | 36 | 37% | 0.32 |
| 1. **Maintaining practitioner-client collaboration** | | | | | | | | | | | |
| H1 | Talking about expectations | | 167 | 92% | 0.34* | 184 | 94% | 0.53* | 90 | 92% | 0.76 |
| H2 | Talking about resistance to care | | 88 | 49% | 0.18 | 147 | 75% | 0.31 | 73 | 74% | 0.56 |
| H3 | Working on motivation | | 115 | 64% | 0.26 | 160 | 82% | 0.51* | 79 | 81% | 0.74 |
| H4 | Offering emotional support | | 161 | 89% | 0.52* | 189 | 97% | 0.72* | 84 | 86% | 0.83* |
| H5 | Working on quality of relationship | | 159 | 88% | 0.41* | 177 | 91% | 0.67* | 80 | 82% | 0.85* |
| H6 | Evaluating relationship | | 134 | 74% | 0.24 | 165 | 38% | 0.36 | 79 | 81% | 0.61 |

*One of ten elements with largest share in this profile

Note. Relative score ranges from 0 (element had small share in overall intervention) to 1 (element had large share).
