# Supplementary material for: Toward tailored care for families with multiple problems: A quasi‐experimental study on effective elements of care
Source: Fam Process. 2021 Dec 21;61(2):571–90. doi: 10.1111/famp.12745 (PMC9305733; doi:10.1111/famp.12745)
Supplement: Supplementary file 4 — Table S3 [file FAMP-61-571-s003.docx]

Supplemental Table 3. Standardized Effects of Practice Element Profiles and Program Elements on Intervention Outcomes in Subgroups: Results of Multiple group Chi-Square Difference Tests

|  | Social contacts** | | Parenting stress** | | Internalizing problems** | | Externalizing problems** | |
| --- | --- | --- | --- | --- | --- | --- | --- | --- |
|  | T1 | T2 | T1 | T2 | T1 | T2 | T1 | T2 |
|  | β | β | β | β | β | β | β | β |
| **Practice elements profiles**  Explorative/supportive | | | | | | | | |
| Combined | | | | | | | | |
| With child intellectual disabilities | 0.242* | 0.218 | -0.070 | -0.261** | -0.069 | 0.080 | -0.137 | 0.206 |
| Without child intellectual disabilities | 0.044 | -0.141 | 0.088 | 0.046 | -0.031 | 0.042 | -0.106 | 0.030 |
| With child psychiatric problems | 0.126 | 0.026 | 0.105 | 0.120 | 0.119 | 0.028 | -0.008 | -0.165 |
| Without child psychiatric problems | 0.079 | -0.112 | 0.044 | -0.109 | 0.116 | 0.040 | -0.020 | 0.082 |
| With parental psychiatric problems | 0.269*^a^ | 0.104^a^ | 0.092 | -0.041 | 0.084 | 0.032 | -0.014 | 0.144 |
| Without parental psychiatric problems | -0.079^b^ | -0.310*^b^ | 0.001 | -0.007 | 0.071 | 0.372*** | -0.048 | 0.199* |
| Action-oriented | | | | | | | | |
| With child intellectual disabilities | 0.124 | 0.038 | 0.038 | -0.205* | -0.086 | -0.188* | -0.171 | 0.337** |
| Without child intellectual disabilities | -0.041 | -0.081 | 0.088 | 0.059 | 0.068 | -0.007 | 0.021 | 0.035 |
| With child psychiatric problems | 0.115 | 0.083 | 0.094 | 0.165 | 0.164 | 0.055 | 0.087 | -0.025 |
| Without child psychiatric problems | -0.049 | -0.061 | 0.089 | -0.033 | 0.082 | -0.006 | 0.058 | 0.223* |
| With parental psychiatric problems | 0.095 | 0.001 | 0.102 | 0.002 | 0.111 | 0.041 | 0.075 | 0.209 |
| Without parental psychiatric problems | -0.051 | -0.143 | 0.016 | 0.044 | 0.124 | 0.127 | 0.059 | 0.110 |
| **Program elements**  Number of visits | | | | | | | | |
| With child intellectual disabilities | -0.223 | # | 0.095 | # | 0.203 | # | 0.295* | # |
| Without child intellectual disabilities | 0.064 | # | 0.044 | # | -0.031 | # | 0.018 | # |
| With child psychiatric problems | -0.028 | -0.131 | 0.121 | 0.239** | 0.028 | 0.160* | 0.181 | 0.137 |
| Without child psychiatric problems | 0.037 | -0.186 | 0.087 | 0.012 | -0.053 | -0.031 | -0.055 | 0.014 |
| With parental psychiatric problems | 0.065 | -0.262 | 0.127 | 0.113 | -0.038 | 0.000 | 0.134 | 0.060 |
| Without parental psychiatric problems | 0.056 | -0.255* | 0.142* | 0.123 | 0.138 | 0.177 | 0.114 | 0.039 |
| Duration of visits | | | | | | | | |
| With child intellectual disabilities | -0.315***^a^ | # | 0.017 | # | 0.106 | # | 0.111 | # |
| Without child intellectual disabilities | -0.039^b^ | # | 0.058 | # | -0.035 | # | 0.012 | # |
| With child psychiatric problems | -0.058 | 0.152 | 0.105 | 0.004 | 0.033 | -0.046 | 0.067 | 0.134 |
| Without child psychiatric problems | -0.099 | -0.161 | -0.084 | 0.142 | -0.017 | -0.119 | -0.025 | -0.190 |
| With parental psychiatric problems | -0.003 | 0.114^a^ | 0.051 | 0.078 | -0.024 | 0.029 | 0.055 | -0.038 |
| Without parental psychiatric problems | -0.153* | -0.313*^b^ | 0.056 | -0.013 | 0.040 | 0.025 | 0.120 | 0.152 |
| Telephone contacts | | | | | | | | |
| With child intellectual disabilities | 0.282* | # | -0.139 | # | -0.088 | # | 0.023 | # |
| Without child intellectual disabilities | 0.100 | # | -0.081 | # | 0.020 | # | 0.018 | # |
| With child psychiatric problems | 0.076 | 0.128 | -0.363**^a^ | -0.568***^a^ | -0.127 | 0.344 | -0.002 | 0.066 |
| Without child psychiatric problems | 0.234* | 0.026 | 0.014^b^ | -0.025^b^ | 0.185 | -0.281 | -0.022 | -0.061 |
| With parental psychiatric problems | 0.119 | 0.017 | -0.010 | -0.089 | 0.180 | 0.012 | 0.184 | -0.018 |
| Without parental psychiatric problems | 0.502*** | 0.263 | -0.264* | -0.110 | -0.149 | -0.192 | -0.109 | -0.019 |
| Intervision | | | | | | | | |
| With child intellectual disabilities | 0.061 | # | -0.175 | # | -0.402* | # | -0.382* | # |
| Without child intellectual disabilities | 0.064 | # | -0.058 | # | -0.190* | # | -0.096 | # |
| With child psychiatric problems | 0.126 | -0.069 | 0.017 | 0.157 | -0.311* | -0.509***^a^ | -0.123 | -0.467** |
| Without child psychiatric problems | -0.058 | -0.021 | -0.123 | -0.020 | -0.219* | -0.164^b^ | -0.176 | -0.135 |
| With parental psychiatric problems | 0.300**^a^ | 0.049 | -0.165* | 0.020 | -0.255* | -0.162 | -0.143 | -0.306 |
| Without parental psychiatric problems | -0.174*^b^ | 0.120 | -0.018 | -0.050 | -0.288* | -0.371*** | -0.223* | -0.057 |
| Supervision | | | | | | | | |
| With child intellectual disabilities | -0.268 | # | -0.051 | # | -0.019 | # | 0.064 | # |
| Without child intellectual disabilities | -0.202** | # | 0.014 | # | -0.097 | # | -0.143 | # |
| With child psychiatric problems | -0.138 | -0.329**^a^ | 0.138 | 0.131 | -0.125 | -0.171 | -0.210 | 0.172 |
| Without child psychiatric problems | -0.150 | 0.211^b^ | 0.084 | -0.229 | -0.132 | 0.384 | -0.183 | 0.047 |
| With parental psychiatric problems | -0.241* | -0.007 | 0.034 | -0.064 | -0.005 | 0.297**^a^ | -0.106 | 0.437*^a^ |
| Without parental psychiatric problems | -0.383*** | -0.390 | 0.127 | -0.004 | -0.273 | -0.217^b^ | -0.276* | -0.541*^b^ |
| Consultation | | | | | | | | |
| With child intellectual disabilities | -0.042 | # | -0.005 | # | -0.031 | # | -0.113 | # |
| Without child intellectual disabilities | -0.010 | # | -0.004 | # | 0.091 | # | 0.033 | # |
| With child psychiatric problems | 0.043 | -0.195 | 0.002 | 0.047 | -0.053 | -0.298**^a^ | -0.206 | -0.214 |
| Without child psychiatric problems | -0.111 | -0.402*** | -0.192* | 0.085 | 0.121 | 0.090^b^ | 0.120 | 0.074 |
| With parental psychiatric problems | -0.100 | -0.164 | -0.097 | -0.029 | 0.104 | 0.197* | -0.050 | -0.001 |
| Without parental psychiatric problems | -0.027 | -0.336* | -0.078 | -0.064 | 0.089 | -0.042 | 0.112 | 0.184 |

* *p* < 0.05, ** *p* < 0.01, *** *p <* 0.001

** To make maximum use of the available data, we used the full information maximum likelihood (FIML) estimation procedure as implemented in Mplus to deal with missing data on the four outcome measures. Original sample sizes for social contacts were T0 = 349, T1 = 224, T2 = 140, for parenting stress T0 = 382, T1 = 252, T2 = 142 and for both internalizing and externalizing problems T0 = 276, T1 = 196, T2 = 91

Note. Different superscripts (a versus b) indicate significant (*p* < .05) differences between subgroups

#No convergence due to sparse data, and number of parameters to be estimated being greater than number of observations in one group

Note. In these analyses we controlled for the propensity score and baseline scores on social contacts, parenting stress, internalizing- and externalizing problems

Note. Profile 1 (explorative/supportive profile) was considered to be the reference group
